# 26 Clinical Competencies for the Burn Speech-Language Pathologist: A Multidisciplinary Development and Consensus Study

**DOI:** 10.1093/jbcr/iraf019.026

**Published:** 2025-04-01

**Authors:** Nicola Clayton, Hadley Regal, Tiffany Mohr, Lori Arguello, Kathleen Kerr, Matthew Godleski

**Affiliations:** Concord Repatriation General Hospital; University of Utah Health Burn Center; Orlando Regional Medical Center; University of Texas Medical Branch; University of Texas Medical Branch; Sunnybrook Health Sciences Centre

## Abstract

**Introduction:**

Clinical competency guidelines provide a benchmark to promote the provision of standard care to ensure safe and optimal patient management. Whilst nationally agreed clinical competencies are available for other specialized areas of Speech Language Pathology practice, none currently exist for burn injury. Therefore, we aimed to develop a competency tool specific to the burns Speech Language Pathologist (SLP).

**Methods:**

Spearheaded by the American Burn Association (ABA) Rehabilitation Committee, a group of recognised burn SLPs, Burn Certified Physical and Occupational Therapists and a Burn Physiatrist used a staged approach to (1) synthesize current practice guidelines from a number of national and international burn centers, (2) employ modified Delphi methodology, and subsequently (3) facilitate and participate in expert consensus meetings, to develop and refine a burn SLP competency tool. The previously published Burn Rehabilitation Therapists Competency Tool (Version 2), endorsed by the ABA, was used as a framework.

**Results:**

Eighteen expert multidisciplinary burn clinicians across 14 medical centers, representing three countries (USA: eight states, Canada: three provinces, Australia: one state) contributed to the development and refinement of the Burns SLP competency tool. A steering committee of five SLPs and the burn physiatrist identified 103 competency statements across 15 core clinical domains relevant to the Burns SLP. These were presented in the Delphi Round 1; 18 participants voted and following a consensus meeting, the tool was refined with statements and domains revised, merged and/or new items created as indicated. Two further Delphi Rounds followed by consensus meetings are currently in process. The final tool will comprise multiple domains (each containing knowledge and application competency statements) tailored specifically to the SLP skill set within adult and pediatric burn injury populations, across the continuum of care from acute through to rehabilitation. The tool presents two tiered levels of expertise: Level 1 = minimum level of specialist skill required to manage a severe burn patient, Level 2 = expert level of specialist skill and recognized resource to other SLPs).

**Conclusions:**

This competency tool is the first internationally accepted standard of care for burn SLPs and provides a guideline for performance in managing burn patients throughout the acute and rehabilitative care continuum.

**Applicability of Research to Practice:**

Internationally agreed competencies are needed for the Burns SLP. Development of a clinical competency package through expert multidisciplinary consultation and consensus will guide training, ensure consistency of care provision and benchmarking across facilities with the mutual goal to optimize patient care and outcomes. Once completed, the competencies may serve as a key benchmark to establishing a burn therapy certification for SLPs.

**Funding for the Study:**

N/A

